# Properties and role of interfaces in multimaterial 3D printed composites

**DOI:** 10.1038/s41598-020-79230-0

**Published:** 2020-12-17

**Authors:** Laura Zorzetto, Luca Andena, Francesco Briatico-Vangosa, Lorenzo De Noni, Jean-Michel Thomassin, Christine Jérôme, Quentin Grossman, Anne Mertens, Richard Weinkamer, Marta Rink, Davide Ruffoni

**Affiliations:** 1 Mechanics of Biological and Bioinspired Materials Laboratory, Department of Aerospace and Mechanical Engineering, University of Liège, Quartier Polytech 1, 4000 Liège, Belgium; 2 Dipartimento di Chimica, Materiali e Ingegneria Chimica “G. Natta”, Politecnico Di Milano, Milan, Italy; 3 Center for Education and Research on Macromolecules, University of Liège, Liège, Belgium; 4 Metallic Materials Science Unit, Department of Aerospace and Mechanical Engineering, University of Liège, Liège, Belgium; 5 Department of Biomaterials, Max Planck Institute of Colloids and Interfaces, Potsdam, Germany

**Keywords:** Composites, Mechanical engineering, Bioinspired materials

## Abstract

In polyjet printing photopolymer droplets are deposited on a build tray, leveled off by a roller and cured by UV light. This technique is attractive to fabricate heterogeneous architectures combining compliant and stiff constituents. Considering the layer-by-layer nature, interfaces between different photopolymers can be formed either before or after UV curing. We analyzed the properties of interfaces in 3D printed composites combining experiments with computer simulations. To investigate photopolymer blending, we characterized the mechanical properties of the so-called digital materials, obtained by mixing compliant and stiff voxels according to different volume fractions. We then used nanoindentation to measure the spatial variation in mechanical properties across bimaterial interfaces at the micrometer level. Finally, to characterize the impact of finite-size interfaces, we fabricated and tested composites having compliant and stiff layers alternating along different directions. We found that interfaces formed by deposition after curing were sharp whereas those formed before curing showed blending of the two materials over a length scale bigger than individual droplet size. We found structural and functional differences of the layered composites depending on the printing orientation and corresponding interface characteristics, which influenced deformation mechanisms. With the wide dissemination of 3D printing techniques, our results should be considered in the development of architectured materials with tailored interfaces between building blocks.

## Introduction

Three dimensional (3D) printing, traditionally used to prototype new design concepts and to build components with complex shapes and microstructures, is regarded as a powerful platform to develop heterogeneous composites with tight control not only over the architecture but also on the local material composition^1^. The possibility to combine basic building blocks with dissimilar characters into a single component is indeed a highly effective approach to improve the mechanical performance, to enhance the functionality and to enrich the design space of composite materials^2^. In the research setting, many different routes have been explored to fabricate multimaterial systems with 3D printing^3^. For example, composites with a stiff phase reinforcing a softer matrix can be obtained using printable polymeric inks or resins pre-loaded with metal or ceramics fibers. Different stimuli such as acoustic^4^, magnetic^5,6^, mechanical^7^ or electrical^8^, can be applied to finely tune the local orientation of the fillers and therefore the mechanical behavior of the composite. Alternatively, photocurable polymeric inks can be pre-blended before depositions to fabricate elastomeric materials featuring well-controlled gradients in stiffness, strength and failure strains^9^. At the same time, commercially mature techniques to manufacture multimaterial objects are sparse and mainly based on direct energy deposition and material jetting processes, such as polyjet printing^10^. Although restricted to polymers, the latter is a unique approach to combine constituent materials with elastic properties varying over three orders of magnitude, i.e. from 10^6^ to 10^9^ Pa^10,11^.

**Figure 1:**
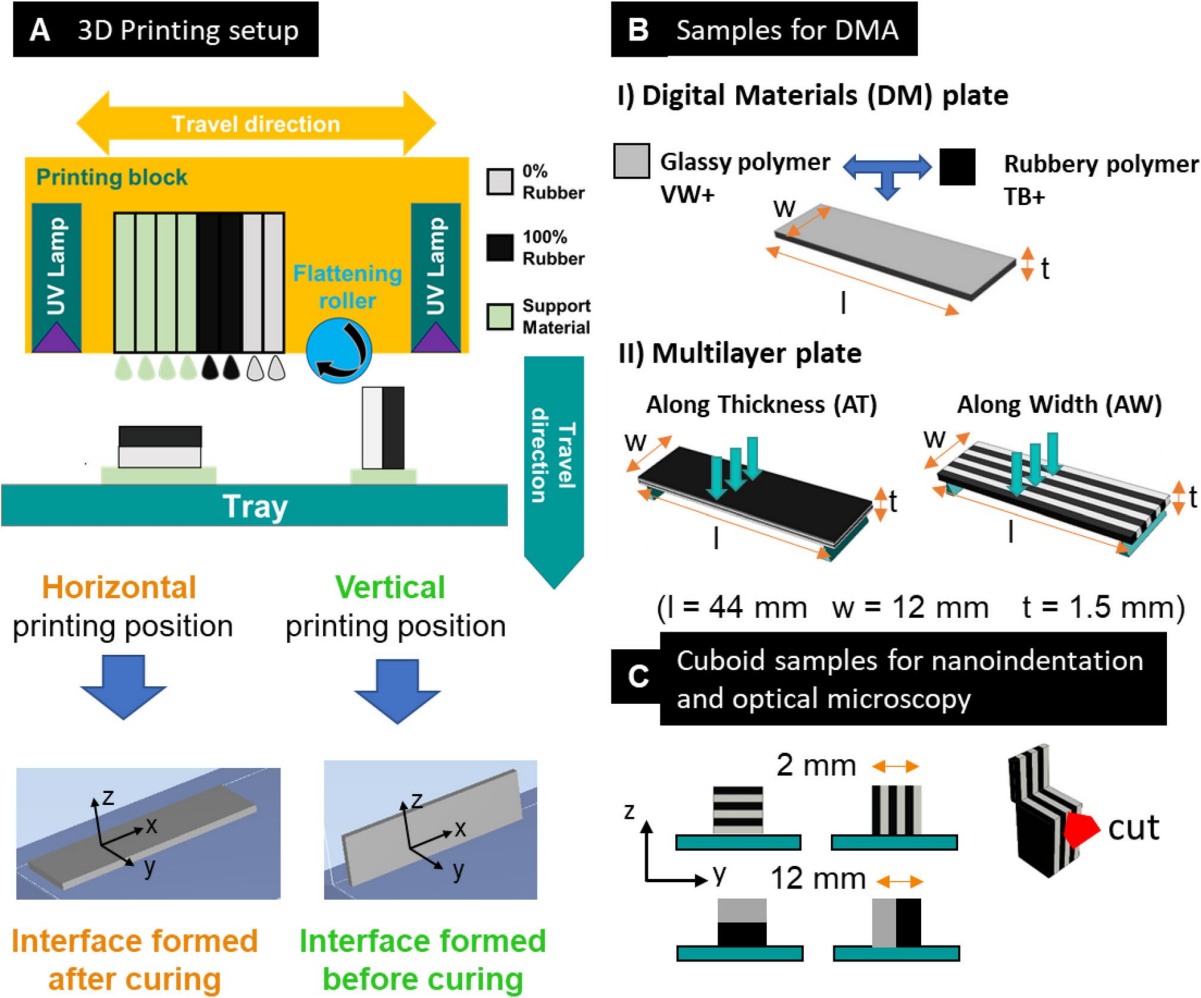
(**A**) Scheme of the polyjet printing process considering bimaterial samples fabricated in horizontal or vertical printing position. The two different printing modalities imply that the bimaterial interface is formed after or before UV curing. (**B**) Plate-like samples consisting of I) digital materials and II) multilayer composites alternating compliant (TangoBlackPlus, TB +) and stiff (VeroWhitePlus, VW +) layers stacked either along the thickness (AT) or the width (AW) of the plate. All samples were assessed with Dynamic Mechanical Analysis (DMA). C) 3D-printed bimaterial cuboid specimens used to characterize interface size with nanoindentation (top) and optical microscopy (bottom). Before nanoindentation, sample surface was smoothed with cryomicrotome: the red arrow indicates the direction of the cut, perpendicular to the interface.

Polyjet printing as implemented in commercial printers is a layer-by-layer method where different photo-curable liquid inks are jetted through several printing nozzles onto a build tray. The deposited micrometer-sized polymer droplets are then leveled off by a roller to prepare the surface for the next layer, and cured by UV light^12^. State-of-the-art commercial polyjet printers (such as those produced by Stratasys, US) use multiple printing heads, which can be loaded with up to five different photopolymers. Each head consists of hundreds of piezoelectric nozzles (aligned along a common direction) depositing liquid drops on pre-established locations while the head block is traveling horizontally (Fig. 1A). Once a layer in the x–y plane is completed, the tray moves down along the vertical z-axis, and the successive layer is built. In multimaterial jetting, the material space can be further expanded by mixing different basic photopolymers directly on the build tray in specific drop-by-drop dithering patterns pre-defined by the manufacturer^10^. This approach enables manufacturing a large variety of blends, referred to as digital materials, with intermediate mechanical behavior^10,11,13^. Even more, it is nowadays possible to assign location-specific material properties (including colors) to each individual voxel within a 3D macroscopic object, enabling the fabrication of complex centimeter-sized heterogeneous structures, referred to as voxel-based materials, with properties spatially tuned at the micrometer level^14–17^. As a consequence, polyjet printing allows prototyping different multimaterial systems, ranging from cellular solids^18,19^ to co-continuous composites^20,21^ and including complex interlocking structures^22^.

The development of novel composites with increased mechanical efficiency can profit from the construction principles observed in natural (or biological) materials. These materials show numerous strategies—refined through the evolution—to generate remarkable functional properties yet using ordinary chemical elements^23^. This is achieved through the arrangement of the basic components into highly complex hierarchical structures^24^, along with a tight control over the interfaces among the different building blocks^25^. In the context of biologically inspired materials, multimaterial printing has been adopted, among other techniques, to fabricate synthetic composites with enhanced and programmable mechanical response^26–28^, as well as to explore the structure–property relationship and the design principles of biological materials^1,29^. Specific examples based on polyjet 3D printing include the spatial arrangements of hard and soft phases typical of mineralized biocomposites such as bone^30,31^ and nacre^32^, the multilayer helix-reinforced structure found in wood^33^ or the plywood fiber-based architecture present in the exoskeleton of crustaceans^34^. In analogy with their biological counterparts^25^, the mechanical response of bioinspired synthetic composites is governed not only by the intrinsic material properties of the individual components (photopolymers) but also by the behavior of the numerous internal multimaterial interfaces^20^.

The relationship between the printing process/parameters and the macroscopic properties of inkjet-based single-material structures has been well-explored^35–38^; however, the knowledge on the local interface properties in multimaterial systems is still rather limited. Considering interfaces, there are essentially two scenarios to be examined, as droplets of distinct photopolymers can get into contact either after or before UV curing. The former happens when one material is deposited onto a previously (at least partially) cured layer of a different photopolymer so that the corresponding bimaterial interface is perpendicular to the vertical z-axis (“horizontal” printing position in Fig. 1A). In the second scenario, the two basic materials are deposited simultaneously on the build tray, and the corresponding bimaterial interface is now parallel to the z-axis of the printer (“vertical” printing position in Fig. 1A). Choosing as basic materials two photopolymers with highly dissimilar properties such as a rigid glassy polymer and a rubbery elastomer (at room temperature), a crucial question is about the size and the properties of the interface in the two scenarios. Despite not having been frequently considered in the relevant literature, this is an important issue in additive manufacturing as interface properties may have a large impact on the mechanical behavior of multimaterial components, including damage and failure^20^. For example, fracture toughness, can vary up to a factor of three for the same bimaterial system depending on the orientation of the interface^39^. The overall strength of multimaterial specimens is governed, not only by printing orientation^40^, but also by the elastic contrast between different polyjet materials. Samples with less contrast between rigid and compliant components have the tendency to fail right at the interface whereas in specimens with large elastic contrast failure occurs more likely in the softest material due to strong constraint effects^41^. Previous results on the interface between 3D printed rigid and elastomeric photopolymers indicate a sharp transition region (about 20 µm) when the two photopolymers get into contact after UV curing^13^. To the best of our knowledge, no data are available regarding the variation of the mechanical properties across the interface in case of simultaneous deposition. However, such information would be essential to design novel heterogeneous multimaterial systems with a well-predictable mechanical response.

In this context, the main objective of the present work is to characterize the mechanical behavior of interfaces obtained via polyjet manufacturing depending on printing modalities, and to show that interface properties have a critical impact on the overall mechanical response of simple multilayer composites. To achieve this goal, we followed a multi-step research strategy combining 3D printing, dynamic mechanical analysis, nanoindentation, optical microscopy and finite element modeling. Firstly, we analyze the dynamic mechanical properties of the so-called digital materials, where rubber- and glassy-like photopolymers are combined according to different volume fractions and following random voxel-based dithering patterns. We investigated digital materials for two reasons: to learn about the degree of miscibility of the two components and to have reference scenarios for interpreting the dynamic behavior of composites with compliant and rigid materials arranged in alternating layers. We then turned the attention to the local interface between compliant and stiff layers: nanoindentation and optical microscopy were used to provide direct evidence of differences in interface properties when changing printing orientation, leading to interfaces formed either before or after UV-curing. Finally, we assessed the impact of interface characteristics on the overall mechanical performance of plate-like multilayer composites having well-defined arrangements and orientations of internal interfaces. Mathematical modeling and finite element (FE) simulations were employed to clarify experimental findings and to further explore the parameter space. Specifically, the purpose of FE simulation was to highlight the role of internal interfaces on the load transfer mechanisms between adjacent layers, considering different assumptions for interface size and elastic properties.

## Study design

Three main types of samples were considered (Fig. 1B and C) to answer specific questions on photopolymer blending, interface properties and interface contribution on the overall mechanical behavior of multilayer systems.

### Digital material samples for investigating photopolymer blending by DMA

To characterize the blending of the two basic photopolymers at different volume fractions, we fabricated plate-like samples of digital materials, where droplets of a rigid glassy polymer (VeroWhitePlus, VW +) and a compliant elastomer-like polymer (TangoBlackPlus, TB +) are combined in voxel-wise 2D dithering patterns after jetting directly on the build tray (see Fig. 1B for sample design and dimensions). The following commercially available digital materials were fabricated, featuring increasing volume fraction of rubber: grey20, grey50, grey60 and shore95, shore70 and shore40. However, as the printer manufacturer does not provide information on the volume fractions of the basic constituents, we analyzed the so-called “assembly maps” which are sequences of images sent to the printer from the controlling software after processing the original CAD model. There, each pixel corresponds to a droplet laid down by the printer nozzle using a specified material (either rubbery or glassy) [28]. Based on these images we calculated the nominal volume fraction of rubbery and glassy photopolymers (Supplemetary Information, Figure S1). All samples were characterized by Dynamic Mechanical Analysis (DMA) without further processing.

### Bimaterial samples to characterize local interface properties by nanoindentation and optical microscopy

Droplets of the two basic photopolymers can get in contact before or after UV curing. To characterize the size of the interface in these two scenarios, we fabricated small samples with alternating layers of VW + and TB + , printed in two different orientations (horizontal and vertical) to form interfaces either during or after UV-curing (see Fig. 1C for sample design and dimensions). Sample surface was smoothed and probed with nanoindentation. Additionally, bigger cubic bimaterial samples were also fabricated along two different orientations (Fig. 1C, bottom), polished and analyzed with optical microscopy.

### Multilayer samples to assess the mechanical impact of internal interfaces by DMA

The dynamic mechanical behavior of bimaterial interfaces as well as the impact of non-ideal interfaces on the overall response of 3D printed composites were studied using simple multilayer plates featuring layers of rigid (VW +) and compliant (TB +) material alternating along the thickness (AT) or the width (AW) of the sandwiches (Fig. 1B). The geometry of the alternating layers of TB + and VW + was set in the CAD model sent to the printer. For the along thickness stacking, we considered from 2 to 8 layers with individual layer thickness ranging from 750 to 187.5 µm, to keep the overall dimension of the plate unaltered. Samples with layers piled along their width had 2 to 12 layers, with individual layer width varying from 6 to 1 mm (Table 1). Due to sample overall geometry, layer dimensions and intra-layer interfacial area were different in the two arrangements, with the AT configuration having larger interfacial area and the AW enabling more layers. All configurations were printed along horizontal and vertical orientations to reproduce the two different types of interface. In the horizontal printing position, samples were printed in the x–y plane with the main dimension of the sample aligned with the x-axis of printer. In the vertical printing position, samples were printed in the x–z plane, again with the main dimension co-aligned with the x-axis (Fig. 1A). Samples featuring layers along their thickness had interface between layers formed after UV curing when printed horizontally and before UV curing if printed in the vertical orientation. The opposite was true for sandwiches with layers of TB + and VW + stacked along their width (Table 1). These samples were characterized using DMA without further manipulation.

**Table 1:**
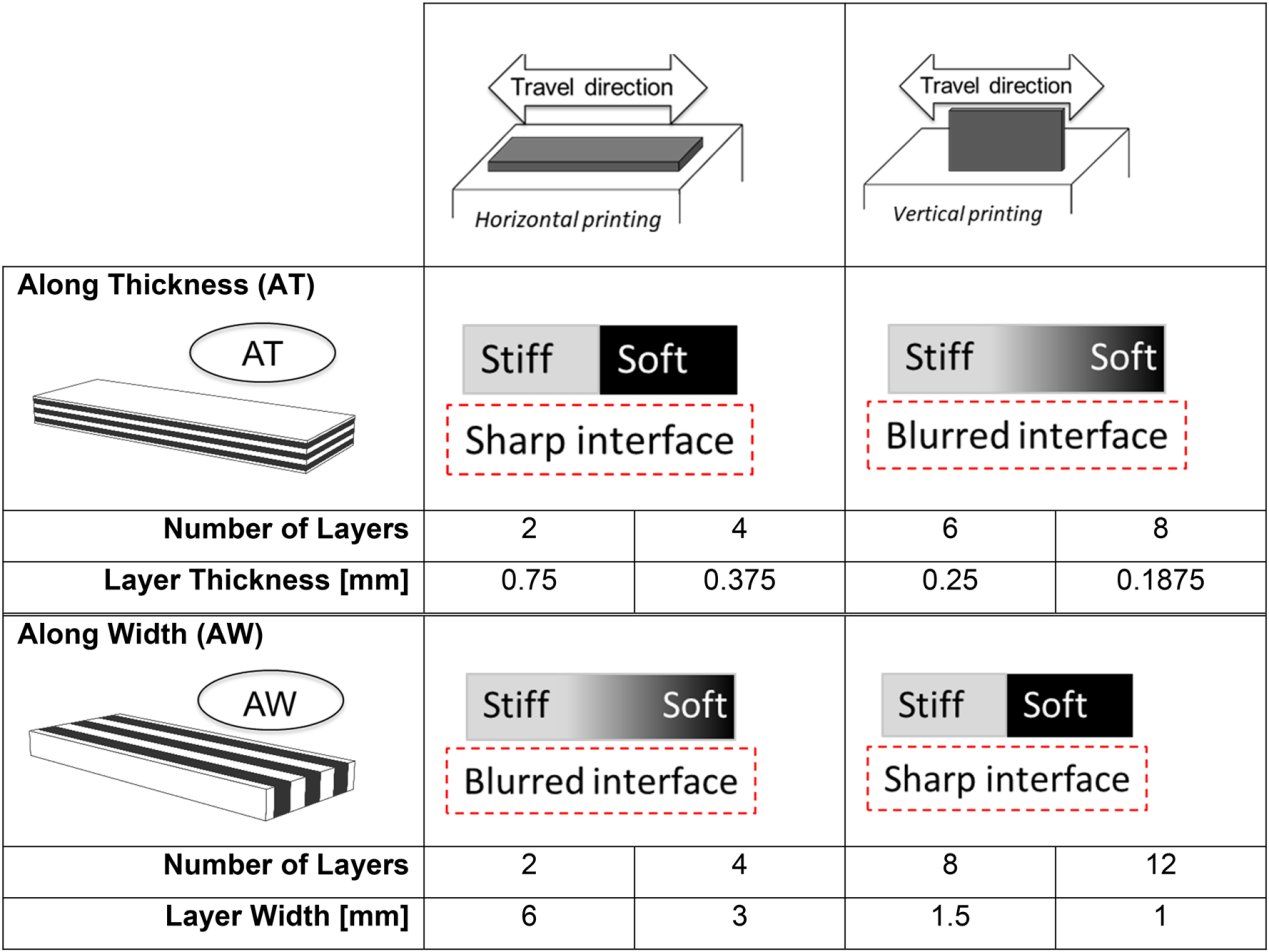
Simplified graphical summary linking layer position (along thickness/width), printing orientation (horizontal/vertical) and corresponding interface (blurred/sharp). The number of layers and corresponding layer dimensions are also indicated.

## Results and discussion

### Dynamic mechanical behavior of digital materials

**Figure 2:**
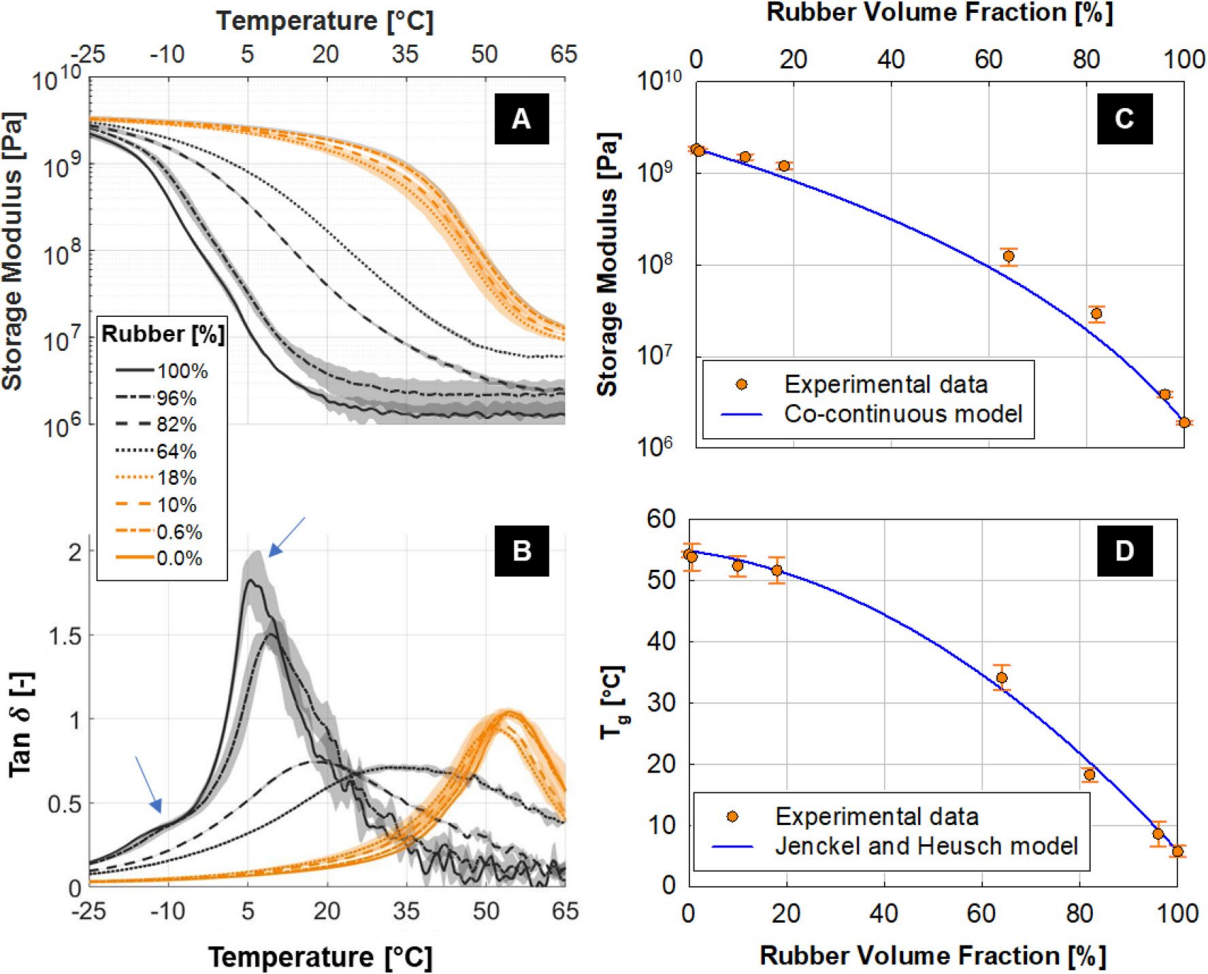
Results of dynamic mechanical analysis (DMA) tests on commercial digital materials printed in horizontal position. (**A**) Storage modulus and (**B**) loss factor (Tan $\delta$) as a function of temperature. Data reported are mean values over three replicates with shaded area representing one standard deviation interval. Blue arrows in (**B**) highlight the individual transition temperatures found in the rubbery photopolymer. (**C**) Experimental mean storage modulus (at 25 °C) compared with the prediction of a simple co-continuous model (Eq. 3). (**D**) Transition temperatures (relative to the predominant peak in Tan $\delta$) along with Jenkel and Heusch fitting model (Eq. 2).

We analyzed the storage modulus (Fig. 2A) and loss factor (Fig. 2B) of the two basic materials (i.e., VW + with 0% rubber and TB + with 100% rubber) and we considered two classes of intermediate digital materials named “overall glassy” with a nominal rubbery content equal to 0.6% (grey20), 10% (grey50) and 18% (grey60), and “overall rubbery” with 64% (shore95), 82% (shore70) and 96% (shore40) rubber. The material VW + , as evidenced by tan $\delta$, had a glass transition at about 54 °C, being glassy (E ≈ 3.5 GPa) and rubbery (E ≈ 20 MPa) below and above this temperature. In the case of TB + , the loss factor (tan $\delta$) indicated two distinct transitions, highlighted by the two arrows in Fig. 2B: the first located at about -12 °C and the second at 6 °C. The storage modulus of TB + changed by more than 3 orders of magnitude in the temperature interval starting well below the first transition and finishing after the second one, i.e. from -25 to 30 °C. Although the chemical composition of the photopolymers is not disclosed by the manufacturer, these results could be explained either by the presence of two distinct phases in the elastomeric polymer or by a secondary beta-transition.

All predominantly glassy samples had a qualitatively similar behavior: only one main transition temperature was evident and its value decreased by about 4 °C when adding up to 18% TB + (Fig. 2B), suggesting a certain degree of miscibility between the two components. At the same time, the storage modulus had a behavior very close to that of pure VW + over the entire temperature range (Fig. 2A). Overall, the properties of the glassy materials seem dominated by VW + , which is in agreement with the experimental evidence of TB + forming soft inclusions into a rigid matrix of VW + . These inclusions have an elongated shape^13^, especially when observed in the y–z plane, probably due to the rapid movement of the printing head while jetting as well as to the anisotropic voxel size (Supplemetary Information, Figure S2). The fact that the transition temperature relevant to the TB + aggregates was not detected may be due to the formation of a non-negligible interface around the inclusions, providing a graded transition between matrix (VW +) and inclusions (TB +) properties. Considering the “rubbery’’ materials, adding small amounts (up to 4%) of VW + to TB + , two low-temperature transitions were present: one again at about -12 °C and the other around 10 °C (Fig. 2B). The latter is slightly higher than the second transition temperature of pure TB + , meaning that probably part of the rubbery phase blended with VW + . Increasing the percentage of VW + (from 4% to 18 and 36%), the loss factor had only a single wide peak, which shifted towards higher temperatures when adding more VW + . Storage modulus and loss factor of the basic rubbery material tango black (TB) and of an additional digital material containing 18% of rubber (grey 60) were previously investigated using DMA within a temperature range from − 50 to 100 °C^42^. In agreement with our findings, the peak in the loss factor shifted towards higher temperatures and got wider when increasing the volume fraction of the glassy component. However, the values of the glass transition temperatures (− 5 °C and 47 °C for TB and grey60, respectively) were lower than in our case. This could be due to differences in testing modality (tension versus three-point bending, frequency of the applied load 0.1 Hz versus 1 Hz) and build orientation. The glass transition temperatures (*T*_*g*_) of the blends as a function of the rubber volume fraction were very well interpolated (R^2^ = 0.99) by the Jenckel and Heusch model (Eq. 2 and Fig. 2D). The small value of the parameter *b* (i.e., 0.78) implies quite a strong interaction between the two photopolymers^43^, likely if there is a similarity in their chemical structure. Indeed the (partial) chemical information provided by Stratasys^44^ supports a certain amount of compatibility between VW + and TB + , both being based on similar chemical components such as urethane and acrylate oligomers^42^. This fact also agrees with a relatively large size of the interface formed before curing and it is in accordance with tan $\delta$ curves featuring only a single peak. Depending on printing orientation a strong adhesion due to blending of the two base materials can take place as confirmed by previous studies on failure behavior of bi-material systems, which highlighted the absence of failure at the interface^30,31^. However, changing printing orientation—and therefore photopolymer mixing—^39^ or reducing the elastic contrast between the stiff and compliant components^41^ could trigger failure right at the bi-material interface.

The relevant storage modulus measured at 25 °C plotted versus rubber content (Fig. 2C), underlines that adding glassy material to a predominately rubbery matrix had a much higher macroscopic effect than the opposite scenario. With 36% of VW + , the storage modulus increased by two orders of magnitude in comparison to pure TB + . Conversely, 18% of TB + to the pure rigid component VW + decreased the storage modulus only by about 18%, which is in line with results from a previous DMA study in tensile configuration^10^. In digital materials the link between elastic properties and composition had a trend similar to a two-phase co-continuous system. This suggests that digital materials, rather than homogenous blends, should be considered as multiphase composites. The conceivable presence of a third phase, caused by blending between randomly placed rigid and compliant voxels, may be responsible for the observed deviations with respect to the co-continuous model. In conclusion, the analysis of the digital materials highlighted the main features of photopolymer blending when voxels of TB + and VW + are randomly combined. In the next sections, local interface properties and blending in more ordered configurations will be explored.

### Size and mechanical properties of multimaterial interfaces

**Figure 3:**
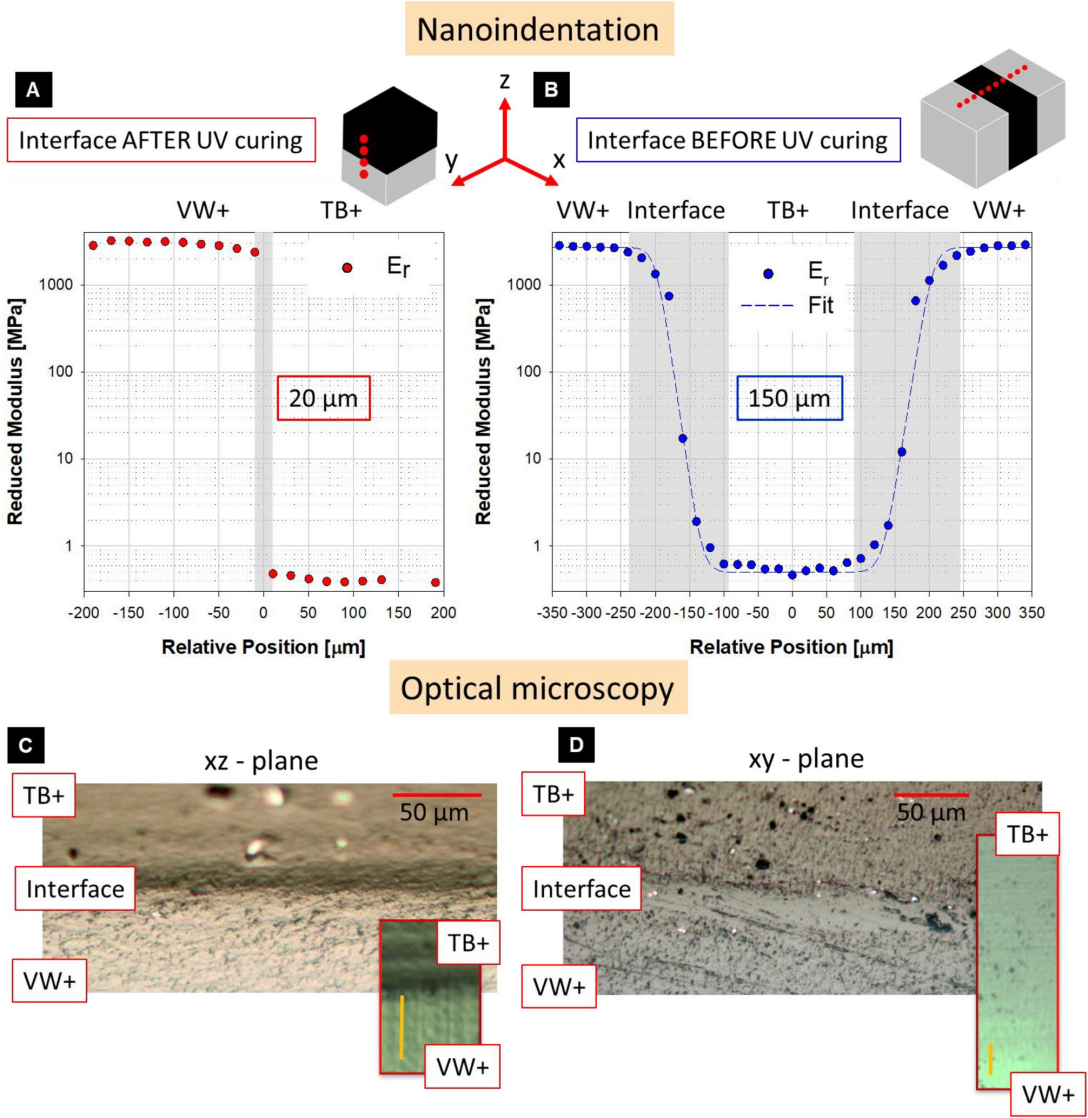
Nanoindentation and optical microscopy results. Spatial variation in reduced elastic modulus (Supplemetary Information, Eqs. S1 and S3) across bimaterial interfaces formed (**A**) after and (**B**) before UV curing. The grey areas in the figures highlight the interface widths in the two scenarios (20 and 150 μm, respectively). At the top, sketch of cuboid samples (TB + and VW + in black and light grey, respectively) showing the printing orientation and indents position. The results are relative to one sample per configuration and to a single indentation line per sample. The other regions which were probed had a quantitatively similar behavior but more data points had to be excluded due to unsatisfactory surface preparation or irregular indentation curves. Inspection of the polished surface of bimaterial samples with optical microscopy confirmed (**C**) sharp and (**D**) broad interfaces for the two printing scenarios. Insets show close-up on the interface region on the cryomicrotome-smoothed samples used for nanoindentation (scale bar 25 μm).

Multimaterial samples possess numerous internal interfaces. A detailed analysis on extension and mechanical properties of such interfaces depending on printing orientation was performed using nanoindentation and optical microscopy. Figure 3A and B show the variations in reduced modulus measured with nanoindentation across the interface between the stiffest (VW +) and the most compliant (TB +) polyjet material provided by the 3D printer. If the photopolymer droplets got into contact after UV curing (i.e. the second material was deposited on an already partially cured first material), the spatial transition in reduced modulus between the two materials was sudden and without any intermediate values between VW + and TB + (Fig. 3A). Interface thickness was therefore smaller than the 20-µm separation between adjacent indents. The size of the interface in this scenario is less than the nominal layer thickness along the axis perpendicular to the interface (z-axis, 800 dpi or ~ 32 µm), suggesting that diffusion between the two different materials is strongly limited by the curing process already occurred in the previously deposited layer. This finding is in agreement with a previous study considering bimaterial samples of VW + and TB + with the interface between the two materials parallel to the build tray and, according to our nomenclature, formed after curing^13^. The authors reported a spatial decrease in elastic modulus of more than three orders of magnitude in about 20 µm when moving from stiff to compliant polymer. However, the absolute value of elastic reduced modulus measured for the two materials (4.46 GPa and 1.08 MPa) were higher than our results. This could be due to differences in sample preparation, nanoindentation probe and load function as well as in the model used to interpret the nanoindentation data (in addition to the intrinsic variability of the photopolymer inks^37^). Optical microscopy analysis of both polished (Fig. 3C) and cryomicrotome-smoothed bimaterial samples (Fig. 3C, inset) confirmed a sharp transition between VW + and TB + , with only minimal mixing at the interface as revealed by a neat separation of the two materials along a fairly flat interface. In contrast, the interface formed before UV curing showed a much broader transition between the soft and the hard component (Fig. 3B). In this scenario, indentation was performed across two consecutive interfaces (i.e., from VW + to TB + and from TB + to VW +) and the spatial variation in reduced modulus was fitted with a sigmoid shaped logistic function^45^:

1 $${E}_{r}\left(x\right)={E}_{0}+\frac{\alpha }{1+{e}^{-\beta \left(x-{x}_{0}\right)}},$$

${E}_{0}$, ${x}_{0}$, $\alpha$ and $\beta$ being the fitting parameters (Supplementary Information, Table S1). The full width of the function was defined as the difference between 0.5% and 99.5% height. The obtained interface width was 145.3 and 152.6 µm when going from VW + to TB + and from TB + to VW + , respectively. This is about 3.5 times bigger than the nominal resolution along the x-direction (~ 42 µm). Here, also optical analysis hinted for a broad interface: considering the polished samples (Fig. 3D), there was not a well-defined transition between TB + and VW + . This is even clearer when looking at the surface of the cryomicrotome-smoothed samples (Fig. 3D, inset): the transition between the darker TB + and the lighter WV + was much more gradual than in the previous scenario and occurred over a region spanning at least 120–140 µm. The optical micrographs of the bimaterial samples differed substantially from those of the commercial blends. The latter were characterized by fiber-like inclusions of the rubbery component into the glassy VW + matrix^13^ (Supplemetary Information, Figure S2). Conversely, even within the broad interfacial region of the bimaterial samples, such inclusions were not detected, suggesting that droplets of the two basic photopolymers have merged well, giving rise to a gradient in optical and material properties.

There are probably two main factors causing interface broadening before curing: droplets are leveled off by a roller (to prepare the surface for depositing the next layer) and this physical process could already facilitate mixing, especially considering the fairly low viscosity of the photopolymers. Second, chemical components of the photopolymers could diffuse and form a blend before being consolidated by UV light. This hypothesis is backed-up by the optical microscopy analysis: as we could not find any signs of interdigitations between adjacent droplets, diffusion mechanisms are likely responsible for the broadening of the interface. Considering the interface as a separate phase with intermediate properties between the rubbery and the glassy components, its effective reduced modulus can be estimated by averaging the experimental points in Fig. 3B within the grey area intervals, giving an average value of about 509.6 MPa. It is worth mentioning that for analyzing the results of nanoindentation, we used well-established methods to extract the reduced modulus from the load–displacement curves. As the conospherical probe of the indenter was much smaller than the printer resolution and, therefore, of the expected length scale of the modulus variation, it seemed reasonable to abstain from additional FE analysis for assessing the accuracy of the indentation results^45^. To conclude, nanoindentation showed clear variations in the properties of interfaces formed before or after UV-curing. Such variations may impact the global mechanical response of 3D printed multilayered systems. This is the focus of the next section.

### Role of interfaces on the mechanical behavior of multilayer composites

#### Load parallel to interfaces: layers along sample width

**Figure 4:**
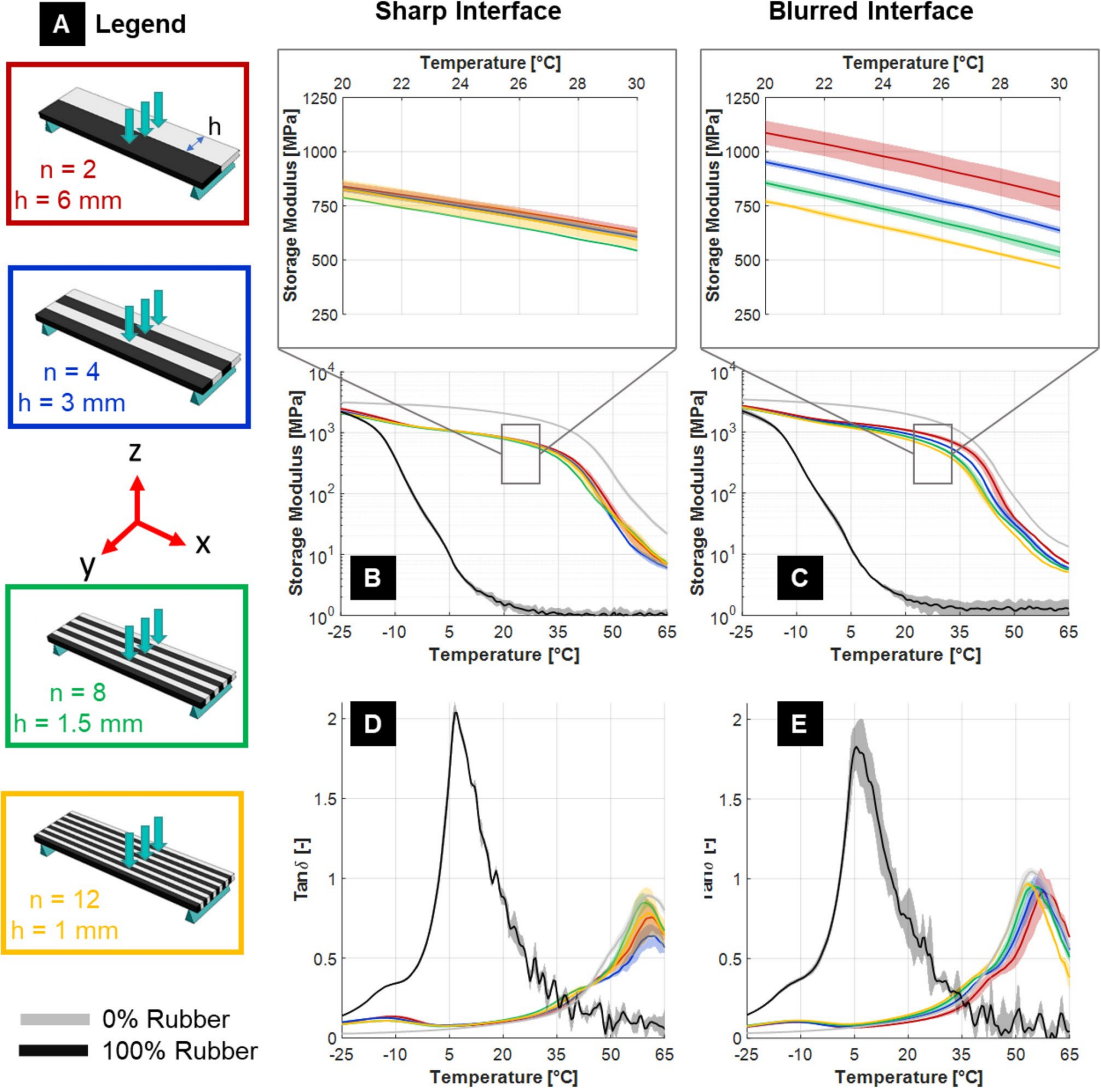
(**A**) Configurations with layers stacked along the width of the samples (n is the total number of layers and h is the layer width). (**B**) Storage modulus and (**D**) loss factor (Tan $\delta$) for composites having sharp bimaterial interfaces (i.e. formed after UV curing). (**C**) Storage modulus and (**E**) loss factor (Tan $\delta$) for composites having blurred bimaterial interfaces (i.e. formed before UV curing). The magnifications show detailed trends in the temperature range 20–30 °C. Data reported as mean value over three replicates with shaded area representing one standard deviation interval. Reference curves for pure materials (black TB + and light grey VW +) are also displayed.

The mechanical response of the composites with alternating compliant/stiff layers stacked along the width of the samples is shown in Fig. 4. Here, interfaces between layers are, in the undeformed configurations, parallel to applied mechanical load (Fig. 4A) and overall interface area is limited, with a value equal to 44 × 1.5 mm^2^ for each layer. When the samples are printed along the vertical orientation (Fig. 1A), interfaces form after UV curing and, according to nanoindentation results, have a thickness of less than 20 µm. Since this value is about two-order of magnitude smaller than the thickness of the individual layers (Table 1), such interfaces are referred to as “sharp’’. Conversely, in multilayer samples printed horizontally, interfaces form before curing and have a much larger thickness, i.e. about 150 μm; they are thus named “blurred’ (Table 1). Assuming an ideal zero-thickness interface, the bending stiffness of the composites should not change when increasing the number of layers while keeping the nominal volume fraction of the two components constant. Indeed, this was observed experimentally for samples having sharp interfaces between layers: focusing on a range of temperature away from phase transitions (e.g., 20–30 °C), the storage modulus of the stacked composites was virtually independent from the number of layers (Fig. 4B and inset). The overall behavior of the loss factor (Fig. 4D) indicated a clear peak above 50 °C due to the glassy component. A smaller peak was also observed at − 10 °C: even if this temperature is lower than the *T*_*g*_ of pure TB + , the peak may still be a fingerprint of the rubbery polymer, showing a slightly different response when combined with VW + (perhaps due to confinement effects).

At variance with these data are multilayer samples with blurred interfaces, showing a distinct trend of decreasing apparent bending modulus with increasing number of layers (Fig. 4C and inset). For example, the storage modulus at 25 °C dropped by 35% when the number of layers increased from 2 to 12. In this configuration, the interface region between the layers is likely to be significantly thicker, accounting for a larger fraction of the overall sample volume. Being the average elastic modulus of the interface (about 500 MPa according to nanoindentation) less than the arithmetic mean of the moduli of the alternating layers (~ 0.93 GPa, calculated using data of Fig. 2C), a decrease in bending stiffness with increasing number of layers is indeed expected. The analysis of tan $\delta$ revealed, in addition to the two already observed main peaks, a “shoulder” to the left side of the higher temperature peak, at about 40 °C (Fig. 4E). Such shoulder, which is far more evident in the case of blurred than sharp interface, could be a fingerprint of blend formation between TB + and VW + .

#### Load perpendicular to interfaces: layers along sample thickness

**Figure 5:**
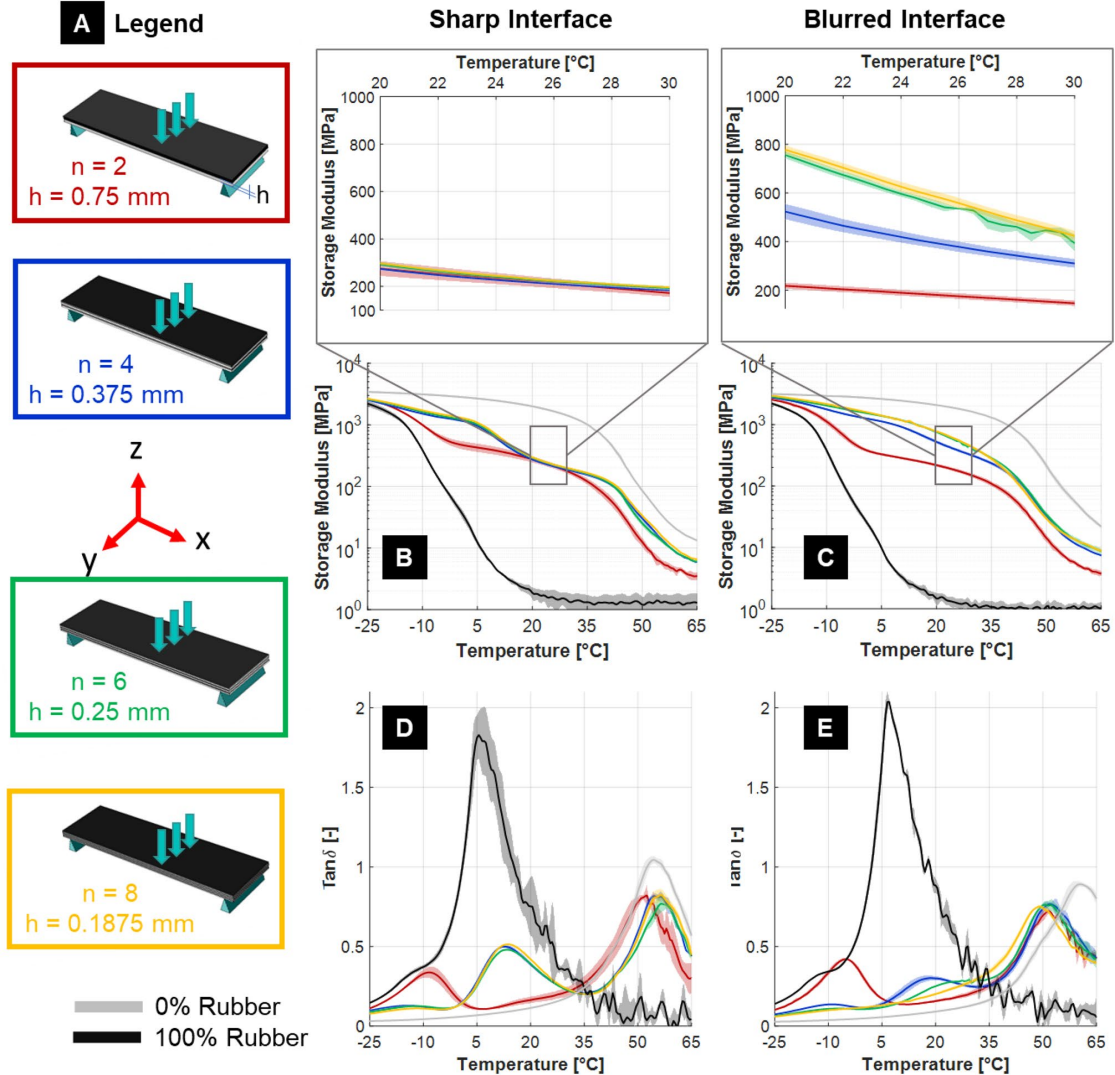
(**A**) Configurations with layers stacked along the thickness of the samples (n is the total number of layers and h is the layer thickness). All samples were tested with the TB + layer always on top. (**B**) Storage modulus and (**D**) loss factor (Tan δ) for composites having sharp bimaterial interfaces (i.e. formed after UV curing). (**C**) Storage modulus and (**E**) loss factor (tan δ) for composites having blurred bimaterial interfaces (i.e. formed before UV curing). The magnifications show detailed trends in the temperature range 20–30 °C. Data reported as mean value over three replicates with shaded area representing one standard deviation interval. Reference curves for pure materials (black TB + and light grey VW +) are also displayed.

In the “along thickness” stacking, interfaces at each soft/rigid layer are initially perpendicular to the applied load (Fig. 5A) and interface area is almost an order of magnitude larger than in the previous scenario (i.e., 44 × 12 mm^2^ for each layer). Here interfaces are sharp when the samples are printed in the horizontal position and blurred when printed vertically (Table 1).

In this configuration, the mechanical behavior measured in three-point-bending should be influenced not only by the inter-layer interface nature, but also by the spatial arrangement of the layers. In fact, according to simple beam theory, redistributing rigid material across the thickness of the plate should increase the bending stiffness of the system (Supplemetary Information, Fig. S5). However, considering the sharp interface scenario and focusing on a temperature range 20—30 °C, the storage modulus had the unanticipated tendency to be rather constant with increasing number of layers (Fig. 5B). Conversely, for the blurred interface, the storage modulus featured a large increase (about 225% at 25 °C) when going from 2 to 8 layers (Fig. 5C). Looking at the initial configuration with two layers, the composites with sharp and blurred interface had a similar response, indicating that the role of the interface was marginal on the overall bending behavior of such bimaterial beams. Conversely, with more than 2 layers, blurred and sharp interface composites showed marked differences, highlighting the critical contribution of interface properties. These experimental observations and the possible presence of non-trivial deformation modes will be further explored using finite element analysis in the next section. Considering the loss factor (Fig. 5D and E), one feature was again the absence of the largest peak observed at about 6 °C for TB + alone, as already noted for all the along width samples. A low temperature peak at about -12 °C was visible for the 2-layer configuration, but as soon as the number of layers increased, this peak became very small. At the same time, a new peak appeared at a position intermediate between the low temperature peak and the high temperature maxima of pure VW + . In the case of sharp interface, this peak occurred at about 13 °C and peak position did not vary when increasing the number of layers (Fig. 5D). As for the blurred interface scenario, the intermediate peak moved towards higher temperature and became broader when having more layers (Fig. 5E). The presence of this peak in the loss factor may be related to the blending of the two photopolymers, causing the formation of a “third’’ material at the interface. The fact that this additional phase became visible in the loss factor is probably due to the geometry of samples with layers stacked along the thickness, together with the regular arrangement of the interfaces. Even if such intermediate peak is present in both sharp and blurred scenarios, its magnitude, position and width are substantially different. Indeed, the amount of blending is expected to be larger with respect to the configuration with layers stacked along the width of the sample, for a given number of layers, the more so considering a blurred rather than a sharp interface. As peak characteristics are likely influenced by the number of layers, the extension of the interface and its location, it is challenging to draw additional conclusions on the blending between TB + and VW + from the analysis of the loss factor. The geometry of the along thickness samples is such that the amount of blending is indeed expected to be larger with respect to along width ones and the complex interplay between the presence of an important interphase, its location and the spatial arrangement of layers is an additional factor that may impact position, magnitude and width of the peak in the loss factor.

In conclusion, the DMA analysis underlined that the mechanical behavior of polyjet based composites is influenced not only by the intrinsic anisotropy of the 3D printing process (as already largely investigated^40,46–48^) but also by the local properties of the internal interfaces. We chose to print all samples with the main dimension aligned along the x-axis of the printer (which corresponds to the traveling direction of the jetting heads). Nevertheless, qualitatively similar findings may have been obtained also by aligning the samples along the y-axis of the printer. The interpretation of the DMA experiments posed several challenges and in the next section, a simple FE model is used to clarify the observed trend in the storage modulus as well as to elucidate the mechanisms of load transfer in the presence of a sharp or blurred interface.

### Finite element simulation of multilayer composites

**Figure 6:**
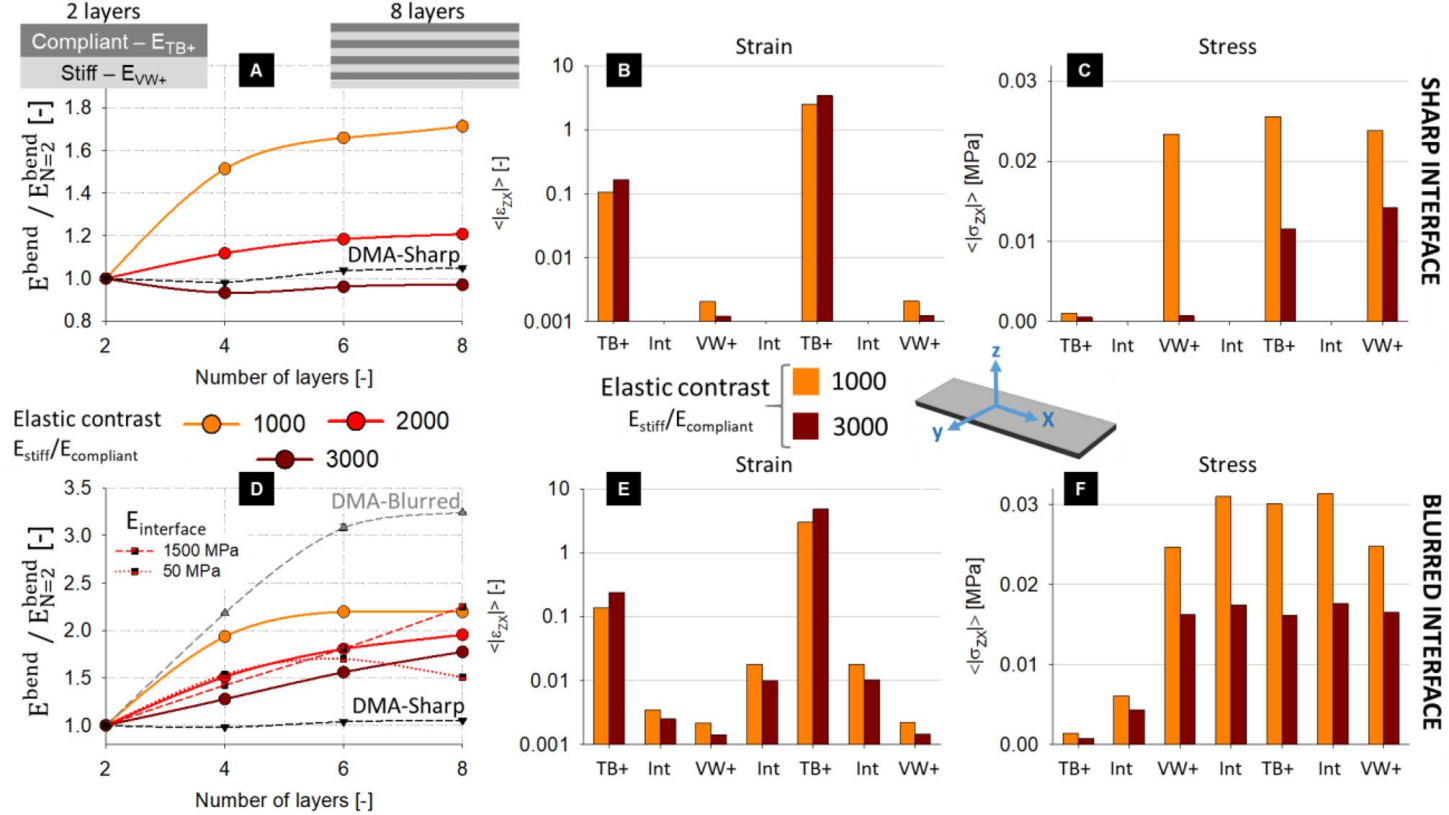
Results of the finite element simulations for the virtual composites with layers stacked along the thickness. (**A**) Apparent bending modulus (calculated with Eq. 4 and normalized by the apparent modulus of the two-layer configuration) for different elastic contrasts between compliant and stiff layers (E_stiff_/E_compliant_ = 1000, 2000 and 3000). In this situation, there was an ideal zero-thickness interface between layers. The experimental behavior obtained by DMA for the sharp interface is also shown. (**B**) Average shear strain and (**C**) shear stress computed within all the alternating layers (for the 4-layer scenario) without interface and assuming two different elastic contrasts (E_stiff_/E_compliant_ = 1000 and 3000). (**D**) Normalized apparent bending modulus assuming a blurred interface between alternating layers (thickness of 150 μm and elastic modulus of 500 MPa). For an elastic contrast of 2000, two additional elastic properties for the interface were considered: 50 and 1500 MPa. Experimental DMA data are shown for both the blurred and sharp interface. (**E**) Average shear strain and (**F**) shear stress computed within all the alternating layers assuming a finite size interlayer interface (thickness 150 μm, elastic modulus 500 MPa).

To gain more insights on the impact of layer arrangement on the mechanical response of multilayer composites we used finite element simulations. Specifically, we calculated the variations in flexural rigidity when subdividing the sandwich into multiple alternating layers, perfectly bonded and stacked along its thickness (Fig. 1B). Firstly, we assumed an ideal zero-thickness interface (corresponding to the sharp interface scenario) and we computed the bending modulus considering different elastic contrast ratios (i.e., E_stiff_/E_compliant_) between stiff and compliant material obtained by varying the modulus of TB + . Increasing the elastic contrast by a factor of three (from 1000 to 3000) switched the behavior of the apparent flexural modulus from a rising (as in the beam model) to a practically constant trend when going from 2 to 8 layers (Fig. 6A). The fact that the bending rigidity does not increase with the number of layers is well in agreement with the experimental DMA results relative to the sharp interface scenario. Such behavior is in contrast with predictions from composite beam theory, always showing a very pronounced increase in flexural modulus with the number of layers (Supplemetary Information, Fig. S5). As in composite beam theory shear mechanisms are neglected, they could be the main reason for the observed trend in the experiments and simulations. Indeed, a detailed analysis of the shear behavior revealed that shear strains were up to three orders of magnitude higher when comparing the compliant layer against the stiff one, and they increased with the elastic contrast in the soft phase (Fig. 6B). The shear behavior observed in the FE simulations may indicate the tendency of the stiff layers to slide with respect to each other through the compliant layer, thus preventing the increase in flexural rigidity predicted by beam theory. Shear stresses were rather similar across the different layers (with the exclusion of the top TB + layer and considering an elastic contrast of 1000), indicating a comparable contribution across the different layers to transmit shear load (Fig. 6C). Increasing the elastic contrast decreased the amount of shear stress in both VW + and TB + regions, meaning that the composites oppose less “resistance’’ to such loading mode. Introducing a finite–size interface between layers with thickness of 150 µm and Young’s modulus of 500 MPa (which may be sensible for the blurred interface scenario, based on nanoindentation results), had a large impact on the bending behavior.

The flexural modulus increased by ~ 77% and ~ 95% when comparing two- to eight-layer configurations for an elastic contrast of 3000 or 2000, respectively (Fig. 6D). Here, the interfacial layers had very low shear strains (Fig. 6E), which were up to two orders of magnitude smaller than the strains measured in the compliant layer and only one order of magnitude higher than the strains in the stiff layer. Therefore, the blurred interface regions reduced the overall volume displaying high shear strains within the composites. They also caused the shear stresses to increase in all layers (Fig. 6F), thus improving the overall resistance of the composites to shear deformations. The effective Young’s modulus of the interface also influenced the apparent bending modulus, although clearly discernible only for eight layers (Fig. 6D). In particular, increasing the modulus of the interface by a factor of 3 (i.e. up to 1500 MPa, which is 3 times higher than measured with nanoindentation) had a somewhat marginal outcome on the overall bending behavior, with flexural rigidity rising by 15% and 11% for an elastic contrast of 2000 and 3000, respectively.

We should mention that, even if the 3D printed polymers might display a non-linear behavior^11,20^, we assumed linear stress–strain constitutive laws and solved the finite element problem for small deformations. The main reason for such simple modeling assumption is that we developed FE models to support the interpretation of DMA testing, which was performed at very small strain amplitude (i.e., 0.1%). Being interested in the behavior at very small strains, our modeling assumptions should match the experimental situation. In fact, simulations were able to capture quantitatively the mechanical response of the sharp interface composites and qualitatively the one of the blurred interface. Nevertheless, experimental results on blurred interface composites showed a somewhat larger increase in bending stiffness compared to FE predictions. A possible way to improve the match between simulations and experiments for the blurred interface case could be to reduce the elastic contrast between TB + and VW + layers, for example by increasing the Young’s modulus of TB + . However, such a reduction would then cause the bending rigidity to increase with the number of layers also in the sharp interface scenario, which is in contrast with the behavior of the experiments. One plausible explanation of the quantitative differences between FE and experiments could be the presence of mechanical gradients within the blurred interface region. In fact, nanoindentation suggests that at the interface, there is a gradual transition in elastic properties between VW + and TB + over several tenths of micrometers (Fig. 3B). Introducing such fine gradients in the FE model, would require an element size not larger than 20 μm (for ensuring mesh convergence), leading to models of almost 100 million elements, which are not only unpractical but also beyond the reach of commercial finite element solvers. In addition to the Young’s modulus, also the Poisson ratio (and shear modulus) may show a graded behavior across the interface, which could impact the overall bending rigidity of the layered composite. It is conceivable that these additional gradients may reduce even more the amount of interlayer shear strains and, at the same time, increase shear stresses, therefore providing a global increase of bending rigidity. It is worth mentioning that the role of interlayers on bending behavior is not only relevant for 3D printed layered systems but has been largely investigated also considering traditional composites, due to the non-trivial slip phenomena occurring at the interface^49,50^.

In conclusion, FE simulations suggested a non-negligible contribution of shear mechanisms in the bending response of the stacked composites and underlined the pivotal role of the interface in the load-transfer ability between different layers.

## Conclusions

This study characterized the properties of bimaterial interfaces in 3D polyjet printing and their impact on the mechanical behavior of multimaterial composites. Following an integrated approach that combined experimental testing at different length scales, analytical modeling and computer simulations, we found that:

(i) In digital materials, the mixing between a stiff (VW +) and a compliant (TB +) polyjet material does not lead to a homogenous blend but has a biphasic nature, as revealed by DMA. Furthermore, digital materials appear as an appropriate model system to study photopolymer mixing and interface effects, being exempt from any phenomenon related to the preferred spatial arrangement of internal interfaces.
(ii) Depending on the printing orientation or sample arrangement, the spatial transition in elastic properties measured with nanoindentation across bimaterial interfaces can either be very sharp (less than 20 µm) or fairly broad (~ 150 µm). The latter is larger than voxel dimensions and underlines a significant inter-diffusion between the two photopolymers taking place before UV-curing.
(iii) Interface characteristics have a critical impact on the mechanical behavior of simple multilayer composites, featuring alternating stiff and compliant layers characterized by a large mismatch in elastic properties. Finite element simulations uncovered the presence of non-negligible shear mechanisms in those heterogeneous composites under three-point bending and clarified the influence of internal interfaces on such deformation mode and on the overall load-transfer mechanisms between layers.

If blending at the interface between the two basic components can affect the macroscopic response of the composites even for a relatively simple geometry (characterized by a small interfacial area) such as the one presently studied, the role of the interface may become even more important when considering more complex geometries, including co-continuous composites or bioinspired interlocking structures, characterized by very large interfacial area. So far, these complex structures have been mainly modeled considering an ideal, zero-thickness interface. The next step could be the explicit inclusion of interface properties (such as those measured here) when modeling polyjet multimaterial composites. Furthermore, we believe the importance of internal interfaces may be even greater when going beyond simple linear elasticity and considering additional irreversible phenomena including material yielding and fracture. Our results also underline the complex interplay between the presence of a thick interface and the spatial arrangement of layers, which is clearly an additional challenge to interpret the (dynamic) bending behavior of the composites. With this in mind, a complementary future work may consider a simpler tensile testing configuration that could help in separating these two effects, in particular when having large interfacial area.

Although previous studies have already investigated several aspects of inkjet-based materials, our work enriches the current literature for the following reasons. Firstly, we extended the characterization of the dynamic behavior of digital materials by testing 6 blends and 2 basic materials over a broad temperature range. Previously, the same blends were tested at a fixed temperature^10^ and only one blend (the glassy material grey 60) and one basic material (TB) were scanned at increasing temperatures^42^. Uncovering the dynamic behavior of the digital materials at different temperatures may facilitate the selection of the blends to be used in specific applications. Furthermore, we described the elastic properties and transition temperatures as a function of the rubbery content using physical and micromechanical models, which can then be exploited to predict the mechanical response of new materials obtained by varying the volume fractions of the basic constituents. Concerning the bimaterial interface, we demonstrated the influence of the printing direction on interface properties. This aspect was already partially investigated but focusing on only one printing orientation^13^ (corresponding to a consecutive deposition of the two materials and therefore to a sharp interface); here we reported novel data on the second relevant printing direction (corresponding to a simultaneous deposition of the two materials causing a very broad interface). The layer-by-layer nature of inkjet manufacturing not only introduces anisotropy in the mechanical properties of single materials, but it also causes strong differences in the behavior of bimaterial interfaces. This is of great importance for the mechanical behavior of multimaterial structures, as highlighted by our results on simple multilayer composites, which give a broader picture on the process-properties relationship in inkjet 3D printing.

To conclude, our results highlight different interface behavior depending on the manufacturing process. We also demonstrated that the role of internal interfaces is critical even if the printed models have features several times bigger than the resolution of the printer. Additionally, the phenomena leading to diffusion of different polymers can be expected for any machine using inkjet technology, irrespective of the printer maker/model or of the particular batch of ink used. Future development of 3D printed architectured materials may capitalize on our findings including them in the design, simulation and printing of optimized multiphase composites.

## Materials and methods

### Sample fabrication

We fabricated samples using a commercial 3D polymer printer (Objet 260, Stratasys, US). The printer allows the simultaneous deposition of micrometer-sized photopolymer droplets of two different basic materials at a resolution of 600 × 300 dots per inch (DPI) in the x–y plane^51^. When printing multiple materials, spatial resolution along z direction is 800 DPI. Bimaterial samples were manufactured combining a rigid glassy polymer (commercial name VeroWhitePlus, VW + , Young’s modulus at room temperature in the range of 2–3 GPa^20^) and an elastomeric polymer (commercial name TangoBlackPlus, TB + , elastic modulus at room temperature of 0.5–1 MPa^11^). All samples were manufactured using the so-called “digital material” mode of the Stratasys printers and choosing the glossy surface finish option. In the digital material printing modality, only one UV lamp is active, with an intensity of about 1780 mJ/cm^2^. UV calibration is performed every 300 printing hours, to ensure each samples received the same amount of UV radiation. Considering the curing time, each layer of the plate-like samples was built in about 10 s, including ink deposition and UV-curing. After the printing, support material was removed using a metal scraper. Sample manufacturing and subsequent storage occurred in a room with controlled temperature and humidity to minimize the influence of these environmental factors.

### Nanoindentation and optical microscopy

Prior to nanoindentation we used a cryomicrotome (EM FC6, Leica, Germany) to obtain a smooth sample surface as the typical mean roughness of polyjet printed parts ranges from about 1 to 18 µm depending on printing orientation and surface finish^52^. The samples were placed in the microtome chamber with the interfaces parallel to the cutting direction (Fig. 1C). Initial cuts were performed with a glass blade and refined cuts with a tungsten knife. The microtome was operated at a temperature of − 60 °C, significantly lower than the rubbery material glass transition (see Results and Discussion). Here, we used samples with five bimaterial interfaces to increase the chances of achieving proper smooth locations to be used for nanoindentation. The surface-smoothed samples were glued onto magnetic disks and positioned on the nanoindenter stage. To facilitate the indentation along a line perpendicular to the interface, samples were manually aligned so that the bimaterial interface matched the grid of the optical microscope of the nanoindenter. Indentation in soft materials can be problematic due to adhesion effects as well as surface identification^53,54^; therefore, we followed a nanoindentation protocol suited to probe both relatively rigid and fairly soft polymers and based on monitoring the full interaction between the sample surface and the nanoindenter probe (Supplemetary Information)^53^. Nanoindentation was performed using a Hysitron TI 950 Triboindenter (Bruker, US) equipped with a conospherical probe with a nominal radius of 5.24 µm and cone angle of 60°. We indented across the interface between soft and hard polymers with spacing between consecutive indents of 20 µm. For each configuration we prepared 2 similar samples and in each samples we performed up to 3 indentation lines in different locations. At each individual indentation point, we measured force-depth curves, which were then analyzed depending on the magnitude of the adhesive pull-off force either with classical Oliver-Pharr theory^55^ (Supplemetary Information, Equation S1) or with the Johnson–Kendall–Roberts adhesion model^56,57^ (Equation S2), to extract the reduced elastic modulus (Supplemetary Equations S1 and S3). The reduced modulus ${E}_{r}$ is a combination of sample indentation modulus and indentation probe modulus: as the elastic modulus of the diamond conospherical probe is much higher than the modulus of the polymeric sample, the sample elastic modulus ${E}_{s}$ can be calculated from the reduced modulus using^55^: $1/{E}_{r}\approx (1-{\nu }_{s}^{2})/{E}_{s}$, with ${\nu }_{s}$. being the sample Poisson ratio. However, considering the uncertainties of the Poisson ratio at the bimaterial interface, we preferred to report the reduced indentation modulus.

Bimaterial samples printed for optical microscopy were polished using increasing grades of SiC paper (Buehler, Carbimet P400, P800, P1200, P2500), including a final step of diamond polishing (Buehler, TexMet C). Polishing was done using a MetaServ 250 (Buehler, Germany) at 300 rpm under constant water cooling. Sample surface was imaged with an optical stereomicroscope (Olympus BX60M, Tokyo, Japan) at different magnifications (× 2.5, × 10 and × 20).

### Dynamic mechanical analysis (DMA)

We tested with dynamic mechanical analysis (DMA) 8 digital materials (including basic rubbery and glassy polymers as well as their blends) and 8 multilayer stacked composites in triplicates for a total of 48 samples. DMA was performed in a three-point-bending configuration with a RSA3 dynamic mechanic analyzer (TA Instruments, US). We scanned a temperature range from − 30 to 65 °C with a heating rate of 5 °C/min. In a pilot study, we compared DMA outcomes considering a slower heating rate of 1 °C/min and found no differences (data not shown). All experimental data were sampled every 1 °C. An oscillatory dynamic strain with frequency of 1 Hz was superimposed to an initial static pre-load of about 500 mN. The amplitude of the dynamic strain was 0.1%: within this small range the material behavior can be assumed linearly viscoelastic. The static load was automatically adjusted during the test to ensure that the three-point-bending fixture was always in contact with the sample. With DMA elastic and damping properties expressed as storage modulus and loss factor (or tan $\delta$) were obtained. The glass transition temperature (*T*_*g*_) was estimated as the temperature corresponding to the main peak in loss factor-temperature curves [32]. Knowing *T*_*g*_ and the volume fractions of the basic constituents of a polymeric blend, the degree of miscibility of the constituents was assessed using the semi-empirical model of Jenckel and Heusch^43^:

2 $${T}_{g}\left({w}_{TB+}\right)={w}_{TB+}{T}_{gTB+}+\left({1-w}_{TB+}\right){T}_{gVW+}+b\cdot w_{TB+}\left({1-w}_{TB+}\right)\left({T}_{gTB+}-{T}_{gVW+}\right).$$

where *T*_*g*_ is the glass transition temperature of a digital material having a given volume fraction of rubber, ${w}_{TB+}$. The transition temperature of the rubbery and glassy components is indicated by ${T}_{gTB+}$ and ${T}_{gVW+}$, respectively. The parameter *b* is inversely proportional to the interaction between the constituents of the blend. We calculated *b* by fitting the experimental data with the least mean square method, using *lsqcurvefit* function in Matlab (MathWorks, US).

A representative value of storage modulus for the digital materials, ${E}_{DM}$, (extracted at 25 °C) was used to explore the relationship between elastic properties and composition. Specifically, experimental results were compared in a first approximation against predictions of a simple co-continuous material model, assuming the combination of a stiff (VW +) with a compliant (TB +) phase^58^:

3 $${E}_{DM}={\left[{{E}_{VW+}}^{1/5}\left(1-{w}_{TB+}\right)+{{E}_{TB+}}^{1/5}{w}_{TB+}\right]}^{5}.$$

${E}_{VW+}$ and ${E}_{TB+}$ are the Young’s modulus of the stiff and compliant phase, which were assigned values of 1.85 GPa and 1.90 MPa, respectively, based on the storage modulus data at 25 °C. ${w}_{TB+}$ denotes again the volume fraction of the rubbery component.

### Finite element analysis

To characterize the impact of interface properties on load transfer mechanisms between neighboring layers we applied finite element analysis (FEA) (ANSYS, Canonsburg, US). Quasi-static three-point bending tests of composites with compliant and stiff layers stacked along the sample thickness (Fig. 1B) were simulated. For the bimaterial interface we considered two different scenarios based on the nanoindentation results: the interface formed after curing was modeled as an ideal zero-thickness interface whereas to describe the interface generated before UV curing we introduced a third region between alternating stiff and compliant layers with thickness and elastic properties directly derived from nanoindentation experiments. The estimated interface thickness was 150 μm and a single value of the elastic modulus was used to model the interface region, obtained by averaging the spatial variations in elastic properties across the interface measured with nanoindentation. The overall dimensions of the virtual composites were the same as the 3D printed samples. We simulated three-point bending tests conditions by imposing a uniform displacement of 0.5 mm along the vertical direction (z-axis) to the center of the top surface of the sample, along its entire width. On the opposite bottom surface, one edge was constrained in all direction to prevent rigid body motions, whereas the other was constrained only along the z-direction. The individual layers were fully bonded, consistently with the fact that in small strains no delamination should occur. The load and the bending support were modeled by constraining nodal displacements. Considering the small strain assumption, effects due to contact should be negligible. All geometries were meshed with three-dimensional 20-node hexahedral elements (element type SOLID186 in ANSYS) with a minimum element size of 50 μm, resulting in $\sim$ 7 × 10^6^ elements. The accuracy of the mesh was verified in a preliminary mesh sensitivity analysis, showing that a 50% reduction in element size changed the output flexural rigidity by less than 0.5%. Finite element models were solved on an Intel Core i7 workstation with 64 GB physical memory using 4 cores and required 20–40 min of CPU time. The main outcome of the simulation was the equivalent bending modulus of the composites obtained considering the geometrical features of the plate-like samples (width *w*, thickness *t* and distance between support *s*), the imposed maximum displacement *d* and the calculate reaction force *F* using the following equation:

4 $${E}^{bend}=\frac{{s}^{3}F}{4wd{t}^{3}}$$

${E}^{bend}$ was calculated as a function of the elastic contrast between the layers (defined as the ratio between the Young’s modulus of the stiff and compliant layer, E_stiff_/E_compliant_), the stiffness of the interface as well as of the number of alternating layers (from 2 to 8). We considered elastic contrasts ranging from 1000 to 3000 obtained by assuming a constant Young’s modulus for the glassy material (E_stiff_ = 3 GPa) and varying the Young’s modulus of the compliant layer (i.e., E_compliant_ = 1, 1.5 and 3 MPa). The interface was described as a homogenous material having a Young’s modulus of 500 MPa; two additional values (50 and 1500 MPa) were also tested to underline the role of interface stiffness on the overall mechanical performance of the composites. Poisson ratio was derived based on manufacturer information and assumed equal to 0.48 for the soft phase and 0.33 for the rigid material and the interface. To characterize the local load transmission mechanism between layers we calculated the mean shear stress and strain within each phase (stiff, compliant and interface), obtained by averaging the absolute value of the shear stress ${\sigma }_{ZX}$ and strain ${\varepsilon }_{ZX}$ with the z-axis being parallel to the applied load and the x-axis aligned with the main dimension of the samples (Fig. 6).

## Supplementary information

Supplementary Information.
