# Supplementary material for: Properties and role of interfaces in multimaterial 3D printed composites
Source: Sci Rep. 2020 Dec 17;10:22285. doi: 10.1038/s41598-020-79230-0 (PMC7747733; doi:10.1038/s41598-020-79230-0)
Supplement: Supplementary file 1 — Supplementary Information. [file 41598_2020_79230_MOESM1_ESM.docx]

Properties and role of interfaces in multimaterial 3D printed composites

*By L. Zorzetto, L. Andena, F. Briatico Vangosa, L. De Noni, J.M. Thomassin, C. Jérôme, Q. Grossman, A. Mertens, R. Weinkamer, M. Rink, D. Ruffoni*

**Supporting Information**

**Digital Materials**

We analyzed the so-called assembly bitmap files, consisting of 2D virtual slices in the x-y plane specifying the spatial position and the type of photopolymer droplets to be jetted (Figure S1A).

| 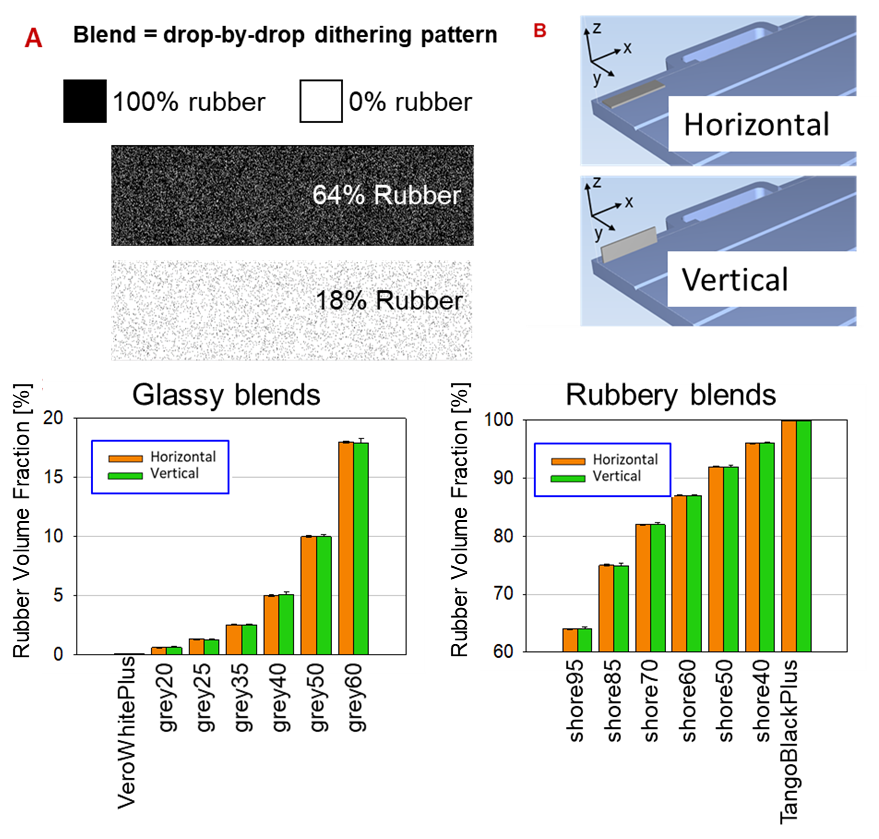 |
| --- |
| Figure S1. (A) 2D assembly maps of two digital materials (overall rubbery with 64% rubber volume fraction and overall glassy with 18% rubber volume fractions) showing the nominal drop-by-drop dithering patterns. (B) Horizontal and vertical printing positions on the printer tray and (C,D) estimated rubbery content of the blends. |

This allowed identifying the nominal material deposition patterns and quantifying the volume fraction of rubbery and glassy components present in each digital material. We considered two different printing orientations for each blend: horizontal, characterized by the smallest dimension of the sample (referred to as thickness) being aligned along the deposition axis (z-axis) and vertical with thickness aligned along the y-axis (Figure S1B). We performed image analysis on all the 12 commercial blends, considering 10 (nonconsecutive) slices for each blend and printing orientation. Figures S1C and D show the corresponding volume fraction of rubbery component in the commercial digital materials, which ranged from 0% to 18% in the overall glassy blends (Figure S1C) and from 64% to 100% in the overall rubbery blends (Figure S1D). No significant differences in composition were found when comparing the two printing modalities, as assessed with a Student’s t-test (p>0.05).

| 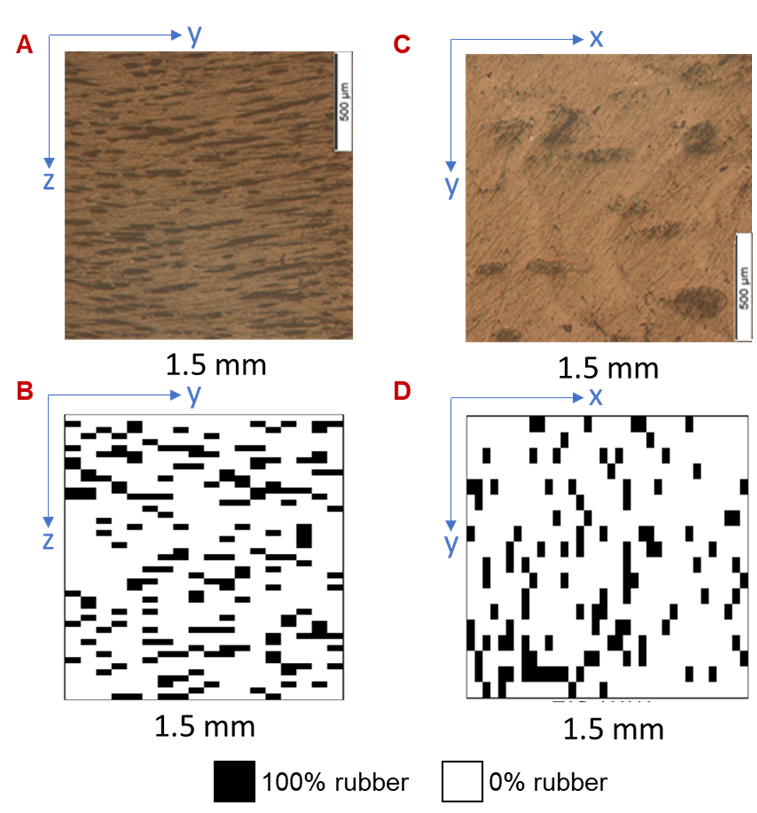 |
| --- |
| Figure S2. Optical micrographs (A, C) and assembly maps (B, D) for a specimen containing 18% of rubber along two different perpendicular planes. |

To investigate to which extent the nominal dithering pattern observed in the assembly map is detectable in the 3D printed samples, we fabricated a rectangular prism (dimensions of 14x10x11.2 mm along x,y and z as done in Ref. ^1^) and we polished the x-y and y-z plane (Figure S2A). Polishing was done following the indications of Buehler (Illinois, US) for polymeric materials (https://www.buehler.com/sumMet.php?material=Polymers), using increasing grades of SiC paper (Buehler, Carbimet P400, P800, P1200, P2500) including a final step of diamond polishing (Buehler, TexMet C). We used a polishing machine (MetaServ 250, Buehler) at 300 rpm under constant water cooling. Sample surface was imaged with an optical stereomicroscope (Olympus BX60M, Tokyo, Japan) at different magnifications (x1, x2.5 and x5). Figure S2 compares the nominal drop-by-drop dithering patterns with the real sample surface obtained after polishing (although not for exactly the same cross section). The binary images were made by processing the assembly maps generated by the 3D printer after processing the CAD model. Specifically, the assembly maps consist of a sequence of images indicating the positon of each voxel to be printed in the x-y plane. These images were combined to generate a single virtual stack using the software ImageJ. After this step, each arbitrary plane in the stack could be easily visualized. In the y-z plane, both the optical micrograph (Figure S2A) and the assembly map (Figure S2B) revealed that inclusions of rubber (TangoBackPlus) in the rigid matrix (VeroWhitePlus) have an elongated fiber like appearance aligned along the y-direction. This is probably due, in addition to the movement of the printing heads and ink rheology as previously suggested ^1^, also to the anisotropic voxel size, with voxel dimension along y (~84 μm) being 2.6 times larger than along z (~32 μm). In the x-y plane, the elongated shape of the inclusion was less evident but still present in the micrograph (Figure S2C) as well as in dithering pattern (Figure S2D). However, the real surface showed inclusions aligned along the x-axis, which corresponds to the movement direction of the jetting head. Conversely, in the nominal assembly map, the elongation lied along the y-direction, again due to anisotropic voxel size (~42 μm and ~84 μm along x and y, respectively). This suggests that the differences between the binary assembly map (Figure S2D) and the real polished surface (Figure 2SC) could be explained based on two competing effects: on one side, the movement of the printing head and the ink rheology tend to elongate the droplets along the x-direction (as seen in Figure 2SC). On the other side, the anisotropic printing resolution would cause the droplets to elongate more along the y-direction (as evident from Figure 2SD). The former is clearly a physical phenomenon not accounted for in the nominal assembly map. In conclusion, rubber inclusions are highly anisotropic objects and their shape depends on the printing process as well as voxel dimensions. Another factor that may potentially influence the observed shape of the inclusion is the degree of mixing of the pigments added to give the black color to the rubbery phase.

**Nanoindentation protocol**

In order to monitor the full interaction between the nanoindenter probe and the sample surface we followed the so-called force curve method (Figure S3) ^2^. In brief, the sample surface was first identified using a traditional approach based on a set force, which in our case ranged from 0.1 to 0.5 µN (depending on the location). The probe was manually withdrawn from the surface to a pre-set distance (lift height) of 10 µm to make sure it was out of contact with the sample and far from the adhesion zone. With a manual re-approach the probe was then moved toward the sample surface till a distance of 2.5 µm above the surface. At this location the displacement-controlled nanoindentation was started tests using an asymmetric trapezoidal load function featuring a loading segment with a maximum displacement of 3.5 µm (corresponding to a total penetration depth in the sample of about 1µm), a hold time of 20 seconds and an unloading segment corresponding to a total displacement of 5 µm. During loading and unloading, the tip was moved at a speed of 100 nm/s ^3^. Such a procedure allowed to capture both the jump-to-contact point (occurring when the probe gets in contact with the sample surface during the approaching phase) as well as the pull-off force, defined as the maximum adhesive force during unloading. If the total pull-off force ($P_{adh}$) was less than 5% the maximum penetration load $P_{max}$, the resulting load-displacement curves were analyzed with the classical Oliver-Pharr method ^4^ after manually re-setting the displacement to zero at the point the load started to monotonically increase, and using the following equation for the reduced modulus ($E_{r}$):

$E_{r}=\frac{S\sqrt{\pi}}{2\sqrt{A\left( h_{c} \right)}}$ . (S1)

The tip area function $A\left( h_{c} \right)$ was calibrated using a polycarbonate standard sample supplied by the manufactured of the nanoindenter (Bruker, US). $S$ is the unloading stiffness calculated using the upper section of the unloading curve, i.e. from 95% to 70% of the peak displacement. The Johnson-Kendal-Roberts (JKR) adhesion model was used if $P_{adh}>5\% P_{max}$ ^5^ after checking that the so-called Tabor parameter ^2^ was much larger than 5. Specifically, we used the following equation to fit the unloading portion (from pull-off to 95% of peak displacement) of the load-displacement ($P-\delta$) curve ^2^:

$\delta=\delta_{contact}+\frac{a_{0}^{2}}{R}\left( \frac{1+\sqrt{1-P/P_{adh}}}{2} \right)^{4/3}-\frac{{2a}_{0}^{2}}{3R}\left( \frac{1+\sqrt{1-P/P_{adh}}}{2} \right)^{1/3}$ . (S2)

The fitting procedure was implemented in Matlab using a nonlinear least-squares solver and allowed the estimation of $\delta_{contact}$ (point of initial contact between the probe and the sample), $a_{0}$ (contact radius between probe and sample at $P=0$ during unloading) and $P_{adh}$ (pull-off force). $R$, the radius of the conospherical probe, was equal to 5.24 µm. These parameters were used to calculate the reduced indentation modulus according to ^2^:

$E_{r}=-\frac{3RP_{adh}}{a_{0}^{3}}$ . (S3)

The minus signed is present as $P_{adh}$ is negative.

| 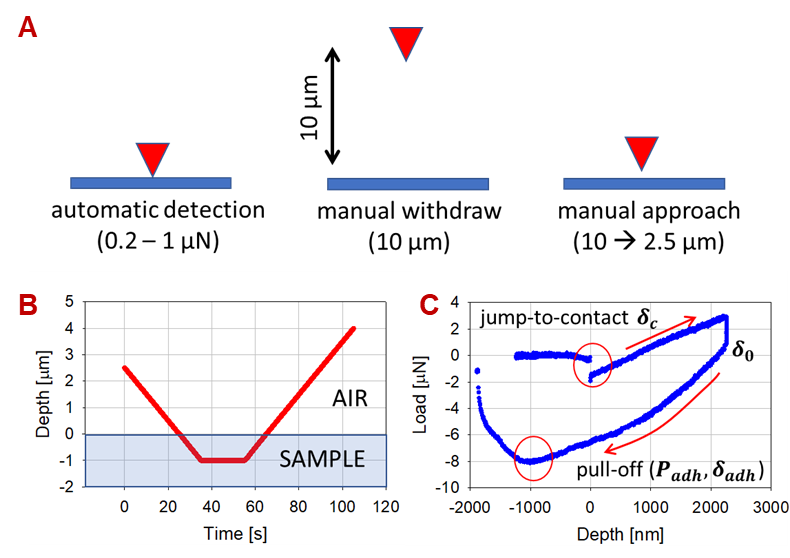 |
| --- |
| Figure S3. (A) Scheme of the manual indentation protocol followed to find the sample surface to indent starting from a given height (2.5 μm) above the surface. (B) Typical trapezoidal displacement controlled load function which allowed a sample penetration of ~ 1 μm. Indentation started out of contact and ended also out of contact. (C) Representative load-depth profile measured in the soft materials (TangoBlack+) with the key parameters used to extract the reduced indentation modulus using the Johnson-Kendall-Roberts model. |

The reduced modulus across the interface between TangoBlackPlus (TB+) and VeroWhitePlus (VW+) in case of interface formed before curing was fitted with a sigmoid shaped logistic function (Equation 1). The corresponding fitting parameters are reported in the following Table S1:

| Interface | $E_{0}$ [MPa] | $\alpha$ [MPa] | $\beta$ [1/μm] | $x_{0}$ [μm] | R^2^ |
| --- | --- | --- | --- | --- | --- |
| VW+ 🡪 TB+ | 0.5679 | 2563.1778 | -0.1308 | -197.4853 | 0.9377 |
| TB+ 🡪 VW+ | 0.5635 | 2467.6041 | 0.1232 | 202.2242 | 0.9081 |

**Equivalent Composite Beam Model**

To facilitate the interpretation of DMA analysis on the multilayer sandwiches, we computed the equivalent bending modulus of idealized multilayer plies using simple composite beam theory ^6^. In short, for the configurations with the layers stacked “along thickness”, the bending stiffness of the composites can be calculated developing an equivalent homogenous beam with a cross-section composed of layers of the same material but having different widths. Specifically, the width of each layer parallel to the bending principal plane (w) is rescaled proportionally to the ratio between a reference elastic modulus (E) and its modulus of elasticity (Figure S4). The reference modulus can be arbitrary chosen. For simplicity, the following example considers 3 layers, but the formulas are easily applicable to an arbitrary number of layers.

| 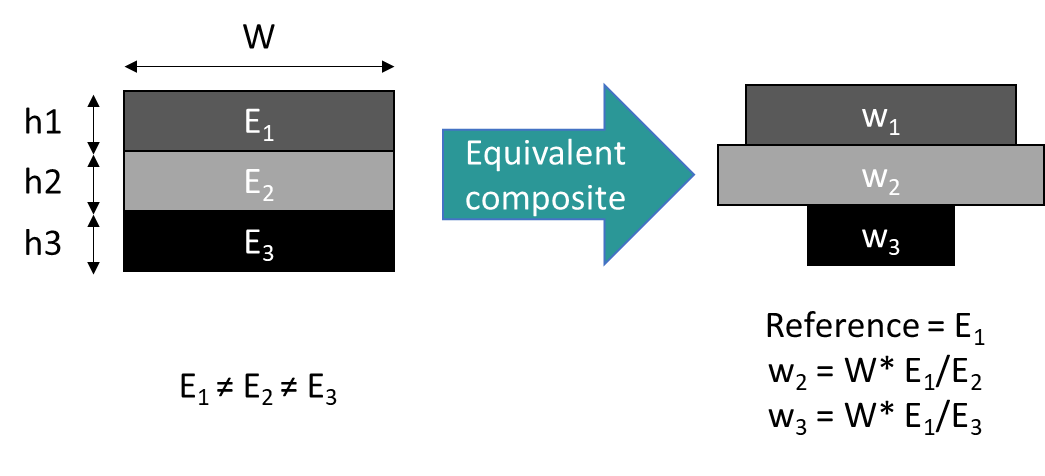 |
| --- |
| Figure S4. Scheme of the equivalent composite beam obtained by compensating the differences in elastic moduli by rescaling the widths of the layers. |

The equivalent cross-section allows to calculate the centroid (y_c_) as follows:

$y_{c}=\left( \frac{h_{1}}{2}+h_{2}+h_{3} \right)A_{1}+\left( \frac{h_{2}}{2}+h_{3} \right)A_{2}+\left( \frac{h_{3}}{2} \right)A_{3}$ . (S4)

Where A_x_ is equal to the area of each cross-section and calculated as $w_{x}\cdot h_{x}$. At this point it is possible to calculate the second area moment (I_eq_):

$I_{eq}= I_{1}+A_{1}\left[ \left( \frac{h_{1}}{2}+h_{2}+h_{3} \right)-y_{c} \right]^{2}+I_{2}{+A}_{2}\left[ \left( \frac{h_{2}}{2}+h_{3} \right)-y_{c} \right]^{2}+I_{3}+{+A}_{3}\left[ \left( \frac{h_{3}}{2} \right)-y_{c} \right]^{2}$ . (S5)

Where I_x_ is second area moment of a single layer calculated as $\frac{w_{x}\cdot h_{x}^{3}}{12}$. The equivalent bending stiffness is calculated as:

$E^{'}=EI = E_{1}*I_{eq}$ (S6)

In this manner, we derived the equivalent flexural stiffness as a function of the elastic contrast between the layers (defined as the ratio between the Young’s modulus of the stiff and compliant layer, E_stiff_ / E_compliant_) as well as of the number of layers. In the model, we assumed an ideal interface of zero thickness between alternating soft and rigid layers. Figure S5 shows the behavior of the flexural stiffness when going from 2 to 8 layers and for different elastic contrasts ranging from 10 to 10000 as estimated with equation S6.

| 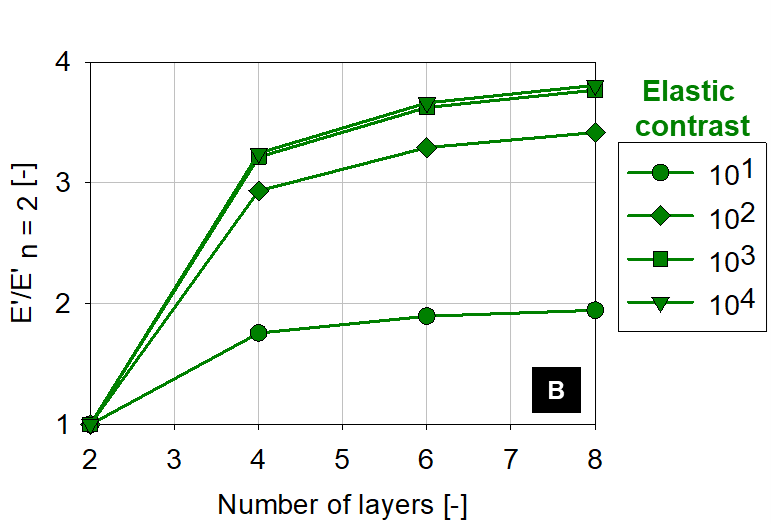 |
| --- |
| Figure S5. Flexural stiffness (normalized by the stiffness of the two-layer configuration) calculated with the composite beam model for an increasing number of layers and considering different elastic contrast between stiff and compliant materials. |

**References**

1 Jochen, M., Diana, C., Manuel, S., Ralph, S. & Kristina, S. Mechanical Properties of Interfaces in Inkjet 3D Printed Single- and Multi-Material Parts. *3D Printing and Additive Manufacturing* **4**, 193-199, doi:10.1089/3dp.2017.0038 (2017).

2 Kohn, J. C. & Ebenstein, D. M. Eliminating adhesion errors in nanoindentation of compliant polymers and hydrogels. *Mech. Behav. Biomed. Mater.* **20**, 316-326, doi:<https://doi.org/10.1016/j.jmbbm.2013.02.002> (2013).

3 Ebenstein, D. M. & Wahl, K. J. A comparison of JKR-based methods to analyze quasi-static and dynamic indentation force curves. *Journal of Colloid and Interface Science* **298**, 652-662, doi:<https://doi.org/10.1016/j.jcis.2005.12.062> (2006).

4 Oliver, W. C. & Pharr, G. M. An improved technique for determining hardness and elastic modulus using load and displacement sensing indentation experiments. *J Mater Res* **7**, 1564-1583, doi:10.1557/JMR.1992.1564 (2011).

5 Tong, K. J. & Ebenstein, D. M. Comparison of Spherical and Flat Tips for Indentation of Hydrogels. *Jom-Us* **67**, 713-719, doi:10.1007/s11837-015-1332-9 (2015).

6 Young, W. C. *Roark&#39;s formulas for stress and strain*. (Seventh edition. New York : McGraw-Hill, [2002] ©2002, 2002).
